# Extramedullary Multiple Myeloma with Hepatic Involvement

**DOI:** 10.7759/cureus.13515

**Published:** 2021-02-23

**Authors:** Manpreet Singh, Harkirat Singh, Phison Pham, Bisharah Rizvi, Ravi Rao

**Affiliations:** 1 Internal Medicine, St Agnes Medical Center, Fresno, USA; 2 Hematology and Oncology, St Agnes Medical Center, Fresno, USA

**Keywords:** multiple myeloma, extramedullary multiple myeloma, hepatic lesions, mm, emm

## Abstract

Hepatic involvement with space-occupying lesions seen in patients with multiple myeloma (MM) is a rare phenomenon. We present two cases of extramedullary multiple myeloma (EMM), with different presentations to highlight the diversity of clinical presentation. Clinically relevant hepatic involvement of myeloma is uncommon and can pose management problems. Hepatic involvement of EMM is indicative of a poor prognosis. Early recognition can help stage and prognosticate the disease.

## Introduction

Multiple Myeloma (MM) is a disease of neoplastic proliferation of plasma cells in the bone that results in the production of monoclonal immunoglobulin [1]. MM is at times preceded by a premalignancy stage known as monoclonal gammopathy of undetermined significance (MGUS) and smoldering MM (SMM), but not all cases go on to develop into MM [1]. The diagnosis of MM is usually made in patients with the well-known CRAB criteria (hypercalcemia, renal insufficiency, anemia, and bone lesions) which is caused by end-organ deposition of free light chains, plasma cell proliferation, and interaction of the plasma cells with the microenvironment.

Extramedullary multiple myeloma (EMM) is an aggressive subentity of MM, characterized by the ability of a subclone to thrive and grow independent of the bone marrow microenvironment, resulting in a high-risk state associated with increased proliferation, evasion of apoptosis and treatment resistance [2]. EMM has been studied since the early 19th century with a wide range of presentations which ultimately depends on which organ system the malignancy invades, with emphasis on tumors that contain reticuloendothelial cells such as liver, kidney, skin, and lymph nodes [1]. EMM is less frequently seen but a very aggressive subset of the disease, with rare cases evolving to or presenting with a leukemic phase termed plasma cell leukemia, which is the most aggressive type of EMM [3]. In patients with confirmed MM, the diagnosis of EMM is usually made by radiological imaging (computed tomography (CT), positron emission tomography (PET)/CT, magnetic resonance imaging (MRI), or ultrasound), biopsy, or at times with physical examination [2]. The mechanisms of extramedullary spread in MM are poorly understood [3]. There is very limited information on the biology of EMM plasma cells but the plasma cells found at these extramedullary sites have a more immature morphology. 

Involvement of the gastrointestinal (GI) system in the course of MM is extremely rare and has been described in the medical literature mainly as case reports of single patients [4]. We present two patients both of whom were diagnosed with EMM with hepatic involvement.

## Case presentation

Case 1

**Figure 1:**
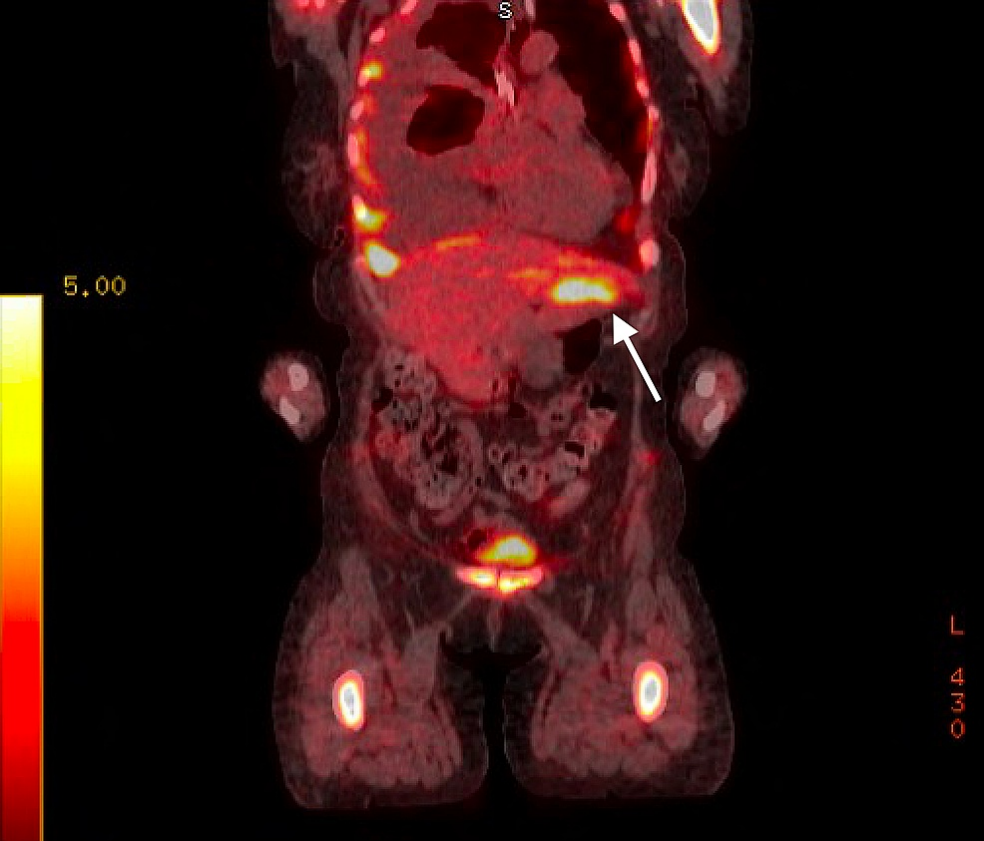
Positron emission tomography (PET) scan revealing multiple hepatic lesions

**Figure 2:**
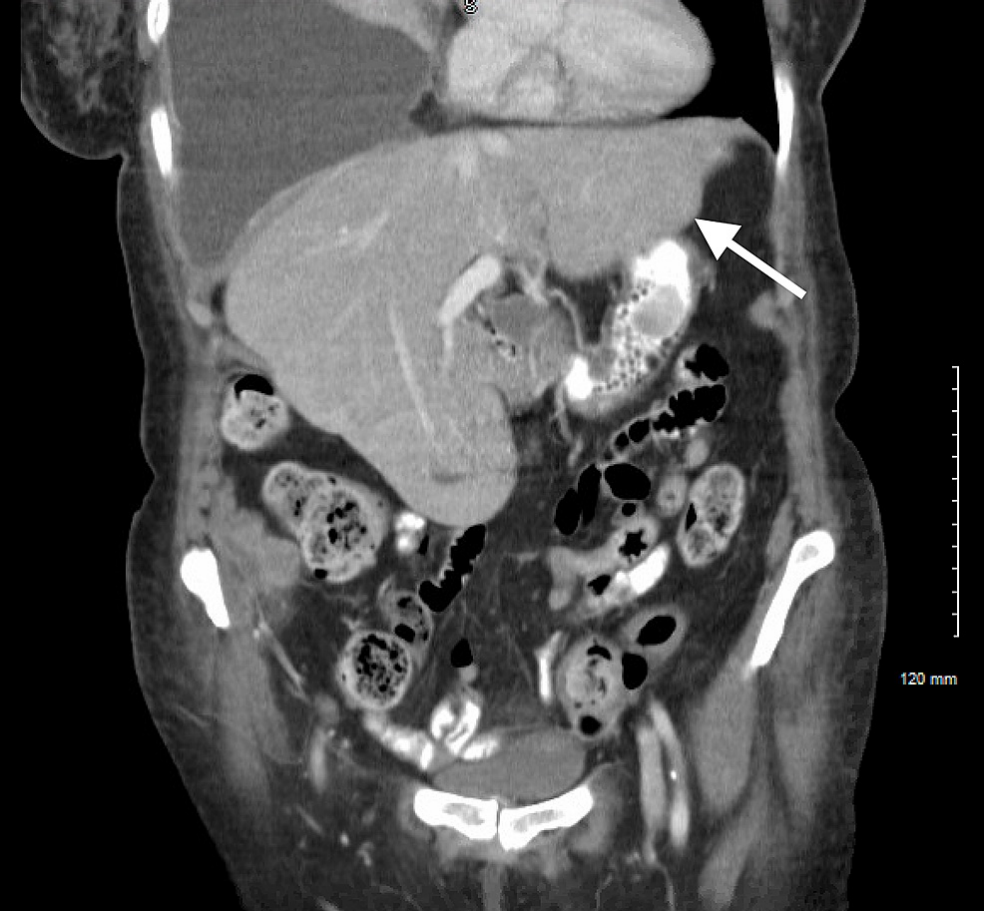
CT abdomen with left hepatic lobe lesion

**Figure 3:**
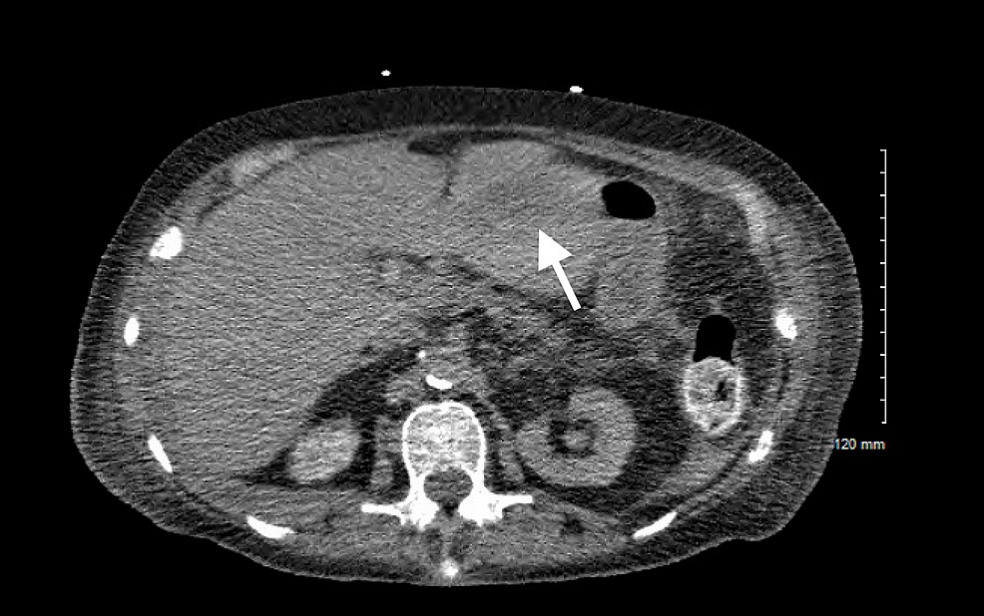
CT abdomen with left hepatic lobe lesion

A 78-year-old woman was diagnosed with MM. At presentation, she had hypercalcemia, bone fractures, anemia, and renal insufficiency (all four CRAB features). Prior to therapy initiation, she had a baseline PET scan that showed multiple hepatic lesions that were PET avid (as seen in Figure 1) and CT abdomen without contrast on admission revealing a poorly defined irregular lesion in the lateral aspect of the left hepatic lobe (Figures 2-3). Labs revealed normal liver enzymes and bilirubin levels. One of these lesions was biopsied and showed diffuse infiltration by cluster of differentiation (CD)138 positive atypical clonal plasma cells. Therapy with carfilzomib, cyclophosphamide, and dexamethasone was initiated. While the kappa light chain values dropped satisfactorily from a baseline of 3645 mg/L to 1014 mg/L in the first three months, her clinical status declined and hospice was initiated. 

Case 2

A 62-year-old woman with a three-year history of nausea, vomiting, weight loss was found to have abnormal liver enzyme elevations. Hepatitis testing was negative. She underwent a liver biopsy. She was found to have clonal plasma cells infiltrating her biliary ducts. A subsequent bone marrow biopsy showed 30% involvement with myeloma. Serum kappa-free light chains were elevated (2368 mg/L). Chemotherapy was initiated, however, she proved to be refractory to multiple lines of therapy. Eventually, after 10 months, she had a partial response to daratumumab, cyclophosphamide, lenalidomide, and dexamethasone. Liver enzymes improved but did not normalize, with alkaline phosphatase remaining persistently elevated. Endoscopic retrograde cholangiopancreatography (ERCP) was negative for any obstruction in the biliary system. Work up for amyloid was negative as well. Given the multiple poor prognostic signs, she is being considered for an autologous stem cell transplantation. 

## Discussion

Involvement of the GI system with an underlying diagnosis of MM is a rare event that has been described in the literature as single case reports [4]. Literature has stated that in up to 40% of MM there is the infiltration of the hepatic system by plasma cells that is clinically silent and usually discovered on autopsy [5]. Liver involvement by MM usually represents a terminal phenomenon. Autopsies performed by Perez-Soler et al. on 128 patients with MM revealed 10 patients with diffuse infiltration of the liver with plasma cells [6]. The histological pattern of liver involvement in MM is seen in the form of light chain deposition disease, extramedullary plasmacytoma, amyloidosis, or a diffuse infiltrative pattern [7]. Patients can also present with non-obstructive jaundice, with elevation in liver function tests (LFT).

The clinical significance of MM with liver involvement at this time is uncertain, with treatment options for hepatic involvement usually requiring systemic therapies [7]. There has been successful treatment reported with combination of chemotherapy and steroid for MM patients with hepatic involvement [8]. Treatment options can become difficult in patients with hepatic dysfunction. The patient in Case 1 presented with normal LFTs and was treated with cyclophosphamide and dexamethasone and had good response with a tremendous drop in her kappa light chain (3645 mg/L to 1014 mg/L), but due to the terminal stage of her MM, her clinical status continued to deteriorate and she eventually was placed on hospice care. The patient in Case 2 did present elevated LFTs and treatment options were limited but ultimately she did respond to the chemotherapy and steroid (daratumumab, cyclophosphamide, lenalidomide, and dexamethasone) and is being considered for autologous stem cell transplantation.

The treatment of EMM with hepatic involvement has no clear treatment guideline due to its rarity, molecular, and proliferative heterogeneity [1]. There are new diagnostic criteria, a new staging system, and more effective treatments being currently being studied [9]. Currently, there are two scoring systems, the Revised International Staging System (R-ISS) and the Mayo Stratification for Myeloma and Risk-adapted Therapy (mSMART 2.0) [6], however, neither one takes EMM involvement. Multidrug combinations (bortezomib, dexamethasone, thalidomide-cisplatin, doxorubicin, cyclophosphamide, and etoposide) can also be utilized for multiple extramedullary myelomas [1], however, defining the best therapeutic regimen remains challenging.

## Conclusions

Our two cases present examples of the spectrum of liver involvement. A review of the literature suggests that liver involvement is a sign of poor prognosis - this was borne out in both our cases. Moreover, liver involvement leads to hepatic dysfunction that limits chemotherapy options due to impaired hepatic function. Liver failure caused solely by MM should be considered in patients with diagnosis of MM and declining hepatic function. For physicians, the presence of nodular hepatic lesions should prompt the clinician to consider hepatic involvement by MM when clinically appropriate. Awareness of this phenomenon can lead to earlier identification of this uncommon presentation of MM.
